# Differences in primary health care use among sub-Saharan African immigrants in Norway: a register-based study

**DOI:** 10.1186/s12913-017-2404-z

**Published:** 2017-07-28

**Authors:** Esperanza Diaz, Vivian N. Mbanya, Abdi A. Gele, Bernadette Kumar

**Affiliations:** 1 Department of Global Public Health and Primary Care, University of Bergen, Bergen, Norway; 2 Norwegian Centre for Minority Health Research, Oslo, Norway; 3 Department of Community Medicine and Global Health, Institute of Health and Society, Faculty of Medicine, University of Oslo, P.O Box 1130, Blindern, 0318 Oslo, Norway; 4 Department of Nursing and Health Promotion, Faculty of Health Sciences, Oslo and Akershus University College of Applied Sciences, Oslo, Norway

**Keywords:** Emigrants and immigrants, Sub-Saharan Africa, Norway, Primary health care

## Abstract

**Background:**

Immigrants’ utilization of primary health care (PHC) services differs from that of the host populations. However, immigrants are often classified in broad groups by continent of origin, and the heterogeneity within the same continent may hide variation in use among immigrant groups at a national level. Differences in utilization of PHC between sub-Saharan African immigrants have not received much attention.

**Methods:**

Registry-based study using merged data from the National Population Register and the Norwegian Health Economics Administration. African immigrants and their descendants registered in Norway in 2008 (36,366 persons) where included in this study. Using χ^2^ test and logistic regression models, we assessed the differences in the use of PHC, including general practitioner (GP) and emergency room (ER) services, and the distribution of morbidity burden for immigrants from Somalia, Ethiopia, Eritrea, and Gambia. For the analyses, we used the number of visits and medical diagnoses from each consultation registered by the physician.

**Result:**

Among the total studied population**,** 66.1% visited PHC within 1 year. The diagnoses registered were similar for all four immigrants groups, regardless of country of origin. Compared to immigrants from Somalia, the age and sex adjusted odds ratios (OR) for use of GP were significantly lower for Ethiopians (OR 0.91; 0.86–0.97), Eritreans (OR 0.85; 0.79–0.91), and Gambians (OR 0.88; 0.80–0.97). Similarly, we also observed lower use of ER among Ethiopians (OR 0.88; 0.81–0.95), Eritreans (OR 0.56; 0.51–0.62) and Gambians (OR 0.81; 0.71–0.92). However, immigrants from Somalia reduced their use of PHC with longer duration of stay in Norway. Differences between groups persisted after further adjustment for employment status.

**Conclusion:**

Despite the similarities in diagnoses among the sub-Saharan African immigrant groups in Norway, their use of PHC services differs by country of origin and length of stay. It is important to assess the reasons for the differences in these groups to identify barriers and facilitators to access to healthcare for future interventions.

**Electronic supplementary material:**

The online version of this article (doi:10.1186/s12913-017-2404-z) contains supplementary material, which is available to authorized users.

## Background

Migration to Europe has increased substantially in the twenty-first century because of economic, political and social factors. In 2010, an estimated 72.6 million migrants lived in the European region, with migrants constituting 8.7% of the total European population [1]. Migrants represent 13.4% of the total population in Norway in 2016, with an additional 2.9% Norwegian-born to immigrant parents. The influx of African migrants to Norway is on the rise, with African- born immigrant population representing 2.2% of the Norwegian population [2]. Somalis are the fourth largest migrant group in Norway, with a population of 41,453 immigrants, while immigrants from Eritrea (23,618) and Ethiopia (10,387) are among the fastest growing migrant groups in Norway. Ghana (2702), Nigeria (2348) and Gambia (1762) are also countries with an increasing immigrant population in Norway [3].

Providing equitable health care services to immigrants remains a challenge to the health care systems. In Norway, the National Health Services are decentralized, with municipalities providing primary health care (PHC). The Norwegian General Practitioners (GP) are the backbone of the PHC and Emergency rooms (ER) are also staffed by GPs out of hours. All immigrants with legal residence permit and asylum seekers are entitled to the same health services as Norwegian-born [4].

The extent of use of GP and ER among immigrants may vary depending on their health care needs, health care seeking behaviours, the organization of health care in their home country, practical barriers to access in the host country, health literacy, migrant’s status, education level and other socioeconomic factors [5–13]. Diverse combinations of these push and pull-factors might influence the use of health care services by immigrants in Norway in different ways.

Immigrants from Africa are often considered a single group because of their geographical location, similar lifestyles, and health problems. Furthermore, in Norway, immigrants from Africa are often grouped with Asian and Latin Americans into a single immigrant population [14–17]. However, the relationship between cultural/social norms and health care utilization patterns seem to differ between nations [18–20]. Prior to migration, sub-Sahara African (SSA) immigrants lived in countries with systems of more self-referral, higher user fees and generally low utilization of health services [21]. Nevertheless, variations in cultural and social norms, prevalence of disease, genetic admixture and health system access in their countries of origin have been described [22–25]. Also, although most immigrants from these countries are refugees, they have different educational and socioeconomic profiles [26]. Thus, once in Norway, their response to a different lifestyle and different health system might vary through different strategies to cope with communication problems, cultural differences, difficulties in their interaction with health systems and providers, and other challenges [10, 27–29].

For these reasons, the heterogeneity among immigrants from Africa should be addressed in order to detect eventual differences among groups and to be able to provide adequate responses to the differing health needs. In this study we aimed to compare the patterns of morbidity burden and the use of PHC services, including GP and ER services, among four of the largest groups of immigrants from SSA countries living in Norway.

## Methods

### Setting and data source

This study includes information from two national Norwegian registers: the National Population Register (NPR) and the Norwegian Health Economics Administration Database (HELFO). These registries were linked by personal identification numbers assigned to all Norwegian citizens and legal immigrants staying in Norway for 6 months or longer. This identification number entitles individuals to access to health care services similarly for immigrants and Norwegians.

Immigrants and their descendants from Somalia, Ethiopia, Eritrea and Gambia registered in Norway in 2008 (*n* = 36,366 individuals), were included in the study. Other SSA immigrant populations in Norway could not be included in the study because the groups were very small. Both first generation immigrants defined as persons born abroad to both parents from abroad and persons born in Norway, with at least one parent from the four selected SSA countries (2nd generation immigrants) were included in the study. Other combinations, like adopted children for the SSA countries, although seldom, were also included in the study to capture disparities among groups.

From the NPR, we obtained socio-demographic variables, including sex, age, marital status, urban or rural settlement, personal income in Norway (in Norwegian crowns), employment status, country of origin, and length of stay in Norway. Age was categorized into four groups for some analyses and length of stay dichotomized by 6 years since registration in Norway. Reason for migration (labour, refugee, family reunification and other reasons) was available only for those who migrated to Norway after 1990.

The HELFO-database contains administrative claims for all patient contacts within the public PHC services including consultations with GPs and ER services. From this register, we obtained information on the number of visits to GPs and ER for each individual in 2008. We used information from consultations both as dichotomous ‘yes or no’ and as numerical variables. Each consultation claim contains at least one medical diagnosis based on the International Classification of Primary Care (ICPC-2) registered by the physician. These ICPC-2 diagnoses were grouped according to the Major Expanded Diagnostic Clusters (MEDC) of the Johns Hopkins University Adjusted Clinical Groups (ACG®) Case-Mix System [30]. The ACG methodology assigns ICPC-2 codes found in claims to one of 27 MEDCs. As broad groupings of diagnosis codes, MEDCs help to remove differences in coding behaviour between practitioners. The ACG System is validated and widely used for research purposes [31].

### Statistical analysis

Descriptive analyses were conducted for socioeconomic variables, use of PHC and MEDCs for the four selected countries. Subject characteristics are presented as means (standard deviation) or percentages for the variables of interest. We then analysed health service use and morbidity burden by age group, gender, and country of origin. Chi-square test and analyses of variance (ANOVA) were used for categorical and continuous variables, respectively, to compare the distribution and differences among immigrants from the four countries. Last, logistic regression analyses were conducted for the outcome dichotomic variables ‘use of the GP’ and ‘use of ER’ to estimate odds ratios (OR) and 95% confidence intervals (CI) for the different countries of origin, using Somalia as a reference. Several models were conducted and results are presented for the unadjusted analyses and the two other models that better explained the use of PHC, one adjusting for age categorized in four groups and gender and the second one for gender, age categorized, and employment status. As interactions were detected between length of stay and country of origin, logistic regression analyses conducted for each of the countries separately and including the length of stay in Norway as an additional variable in the model are presented as a supplementary table. The SPSS 20.0 software package was used for statistical analyses.

## Results

### Demographic characteristic

Table 1 shows the number of subjects, the distribution of the study variables, and the frequency of use of PHC according to the immigrants’ country of origin. The study population comprised of 36,366 SSA immigrants legally registered in 2008 in Norway. Women formed 47% of the studied population and children under 15 years of age were 38.1%. Most immigrants lived in urban areas. Immigrants from Somalia were youngest, the least likely to earn an income and had the highest proportion of unmarried individuals. The mean stay for SSA immigrants was 7.6 years in Norway. With the exception of the Gambian (1.0% refugees), for whom reason for migration was seldom registered, the majority of immigrants were registered as refugees and family reunification. Less than 1.0% in all the groups was labour migrants. Once living in Norway, the proportion of employed SSA immigrants was higher among Ethiopians, Eritreans, and Gambians compared to Somalis.

**Table 1:** Characteristics of the study subjects

Variables	Overall	Somalian	Ethiopian	Eritrean	Gambian	*P*-value
N	36,366	24,253	5631	4483	1999	
Age distribution, %						
0–14	38.1	41.2	34.1	28.5	33.7	<0.001
15–44	52.2	50.8	56.0	56.1	49.9	<0.001
45–64	8.8	7.0	9.6	14.5	16.2	<0.001
≥ 64	0.8	0.9	0.4	0.9	0.2	<0.001
Age, mean (SD)	22.8 (16.0)	21.5 (15.8)	24.0 (15.5)	27.3 (16.7)	25.0 (16.5)	
Women, %	47.0	47.0	46.5	48.7	44.4	0.11
Urban settlement, %	83.3	81.9	86.6	82.6	92.0	<0.001
Marital status, %:						
Unmarried	64.4	65.0	63.1	63.7	61.4	<0.001
Married	24.5	23.8	27.7	27.0	19.2	<0.001
Others (divorced, separated or widow)	11.1	11.2	9.2	9.3	19.4	<0.001
Income, mean [Norwegian crownes]	75,827	53,731	126,980	118,213	104,721	<0.001
Employment status, %						
Outside work force	67.9	73.3	55.3	58.7	58.5	<0.001
Employed	26.0	19.9	40.9	38.0	35.8	<0.001
Self-employed	0.7	0.6	0.8	0.7	1.9	<0.001
Unemployed	3.3	4.0	1.9	1.7	2.4	<0.001
Social welfare beneficiaries	1.8	2.2	1.0	0.9	1.4	<0.001
Immigrants, reasons of migration, %:						
Labour	0.1	0.1	0.4	0.1	0.4	<0.001
Refugee	35.3	37.9	29.7	43.4	1.0	<0.001
Family reunification	25.1	28.8	19.7	11.5	25.6	<0.001
Others	2.2	0.9	7.5	1.5	3.4	<0.001
Reason not specified	37.3	32.3	42.7	43.5	69.6	<0.001
Length of stay in Norway, mean (SD)	7.63 (7.15)	6.61 (5.39)	8.51 (9.07)	9.33 (9.71)	13.74 (8.77)	<0.001
Immigrants background, %						
Immigrant	69.0	71.2	64.4	71.8	48.6	<0.001
Born in Norway with immigrant parent	23.3	26.3	14.2	20.4	18.7	<0.001
Born out of Norway with one parent a Norwegian	0.3	0.0	1.5	0.4	0.3	<0.001
Born in Norway with one parent a Norwegian	5.1	2.4	7.1	5.0	32.4	<0.001
Born out of Norway with both parent Norwegian	2.3	0.0	12.8	2.4	0.0	<0.001
Norwegian nationality, %	56.5	54.2	58.2	55.4	81.5	<0.001
Use of health care services, mean (SD)						
Number of consultations with GP in 2008	2.42 (3.38)	2.48 (3.41)	2.29 (3.30)	2.33 (3.45)	2.14 (3.01)	<0.001
Number of consultations at ER in 2008	0.24 (0.67)	0.27 (0.72)	0.22 (0.61)	0.14 (0.48)	0.19 (0.53)	<0.001

### Use of health care services

A total of 66.1% of all immigrant groups visited either the GP or the ER in 2008, with annual means (standard deviations) of GP and ER visits of 2.42 (3.38), and 0.24 (0.68), respectively (Table 1). The proportion of each immigrant group who used PHC services by age group is presented in Table 2. The use of GP increased with age in all the four countries. Use of GP was similar for the four countries by age group, except for young adults (15–44 years) from Somalia, who used the GP more than those from the other SSA immigrants groups. For all countries, children (0–14 years) and the elderly (over 65 years) used the ER more than the other age groups (15–64 years). Generally, Somalis were over-represented in all age groups at the ER, while Eritreans had the lowest user rates.

**Table 2:** Proportion of use of primary health care services across immigrants’ countries of origin by age group

	Somalia (*N* = 24,253)	Ethiopia (*N* = 5631)	Eritrea (*N* = 4483)	Gambia (*N* = 1999)	Total (*N* = 36,366)	*P* value
General practitioner, %						
Age range: 0–14	58.8	57.4	57.2	55.5	58.3	0.208
15–44	66.8	64.5	61.6	62.7	65.5	<0.001
45–64	71.1	68.1	72.3	73.1	71.4	0.293
≥65	71.8	85.0	63.4	100	71.9	0.193
Total:	63.9	62.5	61.9	62.0	63.3	0.016
Emergency room, %						
Age range: 0–14	17.8	18.4	12.5	15.0	17.3	<0.001
15–44	17.5	15.0	9.8	15.2	15.2	<0.001
45–64	15.9	11.7	10.6	13.0	13.0	0.003
≥65	18.5	10.0	14.6	0.0	17.1	0.567
Total:	17.5	15.8	10.7	14.8	16.3	<0.001

In binary logistic regression analyses, immigrants from Ethiopia, Eritrea and Gambia had significantly lower odds ratios of use of both GP and the ER in 2008 compared to Somalis in unadjusted and adjusted models with the exception of unadjusted analyses of GP use for Gambia (Table 3). Effect modifications between the country of origin and length of stay were however, observed when we included the length of stay in the model (Additional file 1). After adjustment for sex, age and employment status, immigrants from Somalia and Gambia significantly reduced their use of both GP and ER services after 6 years living in Norway while those from Eritrea increased their use of GP but not of ER and Ethiopians did not change their use of PHC with length of stay.

**Table 3:** Use of General Practitioner and Emergency Room services by immigrants’ country of origin. Logistic regression analyses with Somalia as the reference group

	Use of GP (yes/no)			Use of ER (yes/no)		
	OR (95% CI)	*P*-value	Nagelkerke R^2^	OR (95% CI)	Nagelkerke R^2^	*P*-value
Model 1						
Somalia	1		0.017	1	0.007	
Ethiopia	0.94 (0.88–0.99)	0.048		0.88 (0.81–0.95)		0.002
Eritrea	0.92 (0.86–0.98)	0.012		0.56 (0.51–0.62)		
Gambia	0.92 (0.84–1.01)	0.095		0.81 (0.71–0.92)		0.002
Model 2						
Somalia	1		0.025	1	0.011	
Ethiopia	0.91 (0.86–0.97)	0.004		0.89 (0.82–0.96)		0.005
Eritrea	0.85 (0.79–0.91)	<0.001		0.57 (0.51–0.63)		<0.001
Gambia	0.88 (0.80–0.97)	0.010		0.83 (0.73–0.94)		0.005
Model 3						
Somalia	1		0.050	1	0.014	
Ethiopia	0.83 (0.78–0.88)	<0.001		0.85 (0.79–0.93)		<0.001
Eritrea	0.80 (0.74–0.85)	<0.001		0.55 (0.50–0.61)		<0.001
Gambia	0.83 (0.75–0.91)	<0.001		0.80 (0.70–0.91)		0.001

Model 1: unadjusted; Model 2: adjusted for gender and age categorized into four groups (0–14, 15–44; 45–64 and 65+ years of age); Model 3: adjusted for gender, age categorized into four groups (0–14, 15–44; 45–64 and 65+ years of age) and employment status

*OR* Odds ratio, 95% confidence interval

### Diagnoses

**Fig. 1:**
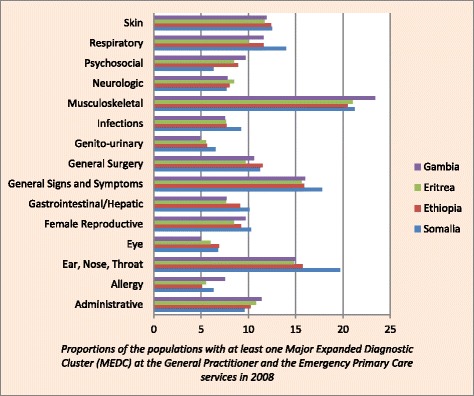
Proportions of the populations with at least one Major Expanded Diagnostic Cluster (MEDC) at the General Practitioner and the Emergency Primary Care services in 2008

Figure 1 represents the proportion of immigrants from each country with at least one MEDC registered in 2008. The most common diagnostic groups among SSA immigrants included musculoskeletal, general signs and symptoms, ear-nose-throat and respiratory related diagnoses. Generally, small differences in diagnoses among immigrants according to the country of origin were detected. Somali immigrants more often than Ethiopian, Eritrean, and Gambian had diagnoses related to ear-nose- throat (19.7% vs 15.7%, 14.8% and 15.0%, respectively); general signs and symptoms (17.8% vs 15.9%, 15.6% and 16.0%, respectively), and respiratory (14.0% vs 11.6%, 10.0% and 11.6%, respectively). Immigrants from Gambia had more often musculoskeletal problems (23.4% vs 20.5–21.2% of all the other groups, respectively).

## Discussion

### Summary of main findings

Our study confirms differences in the use of PHC services across the major four SSA immigrant groups in Norway. Immigrants from Somalia used the PHC services, especially ER services, more than the Ethiopian, Eritrean, and Gambian, although all had relatively similar diagnoses when in contact with the PHC.

In other European countries as well as in Norway, studies have reported differences in the use of PHC across different immigrant populations compared to natives [14, 32, 33]. Overall, our study reports lower mean number of annual visits to the GP but higher to the ER compared to what have previously been reported for immigrants from low income countries in a similar health survey comparing immigrant groups with natives in Norway [34]. As hypothesized previously, differences in the findings could be explained by the pooling of heterogeneous immigrant populations in the same group in the referred study, in which immigrants were classified according to World Bank income categories. Accordingly, another study on ER use using register data from Norway from 2008 showed that immigrants from Somalia more often attended the ER compared to native Norwegians [35].

Somalis in our study used the PHC services more than other SSA immigrants; approximately 15–20% more for GP services and 15–45% more ER after adjustment for age, gender and employment status. Because of the nature of our study, we cannot explain the reasons for the differences in the use of PHC. The higher frequency of PHC use among Somalis compared to the other SSA immigrant groups might be appropriate if it reflects a higher burden of disease. However, once in contact with the PHC, the distribution of the MEDCs in the four SSA groups presented more similarities than differences, suggesting other additional reasons to explain differences in use. The three most common diagnoses reported among all the immigrant populations irrespective of the country of origin were musculoskeletal, general signs and symptoms and ear, nose and throat morbidities, with the latter being most common among Somalis, probably due to the higher proportion of children. However, when compared to native Norwegians, earlier studies have pointed to an overrepresentation of non-specific diagnoses and consultations at night among Somalis at the ER [35]. In contrast to our study, studies in other countries show that immigrants from SSA have a worse health profile compared to other immigrant groups in the same country and as well as the native population [36, 37].

Differences among countries in our study could be explained by the characteristic of the populations, which include the diverse immigrant background of the groups. As more immigrants from Ethiopia, Eritrea, and Gambia had at least one Norwegian parent, they probably encountered less communication challenges and had higher knowledge regarding health care services. Socioeconomic status, with Somalis having the lowest income in Norway, could also partially play a role, although our adjusted model including employment status still showed dissimilarities among groups. Variation in the use of PHC in our study might also be explained by differences in unmet health care needs, formal and informal information about how to access PHC or satisfaction with the health system organization in terms of patients/providers interaction, waiting time to get an appointment or convenience of hours of service. Other individual factors like health literacy, fear of stigma or differences in acculturation and combination of stress related to pre-migration and migration experiences can be differentially distributed between groups [10, 20, 38, 39].

The length of stay is often used as a proxy for acculturation to the new country, and in our study was differentially related to PHC use for the four countries of origin. Duration of stay in immigrants’ host countries tends to improve immigrants’ knowledge of the health care system, language skills and consequently improves utilization of health care services [40]. On the other hand, although immigrants tend to be healthier when they arrive at a new country, which is known as the healthy immigrant effect, their health worsens with time in the new country quicker than the host population [41, 42]. Previous studies show therefore a general increase in the use of health services after some years in the new country [16, 43]. In our study, however, the pattern seems to be reversed for immigrants from Somalia and Gambia. These results should further be studied qualitatively to better understand the underlying causes.

#### Strengths and limitations of the study

Using register data with nationwide coverage is the main strength of our study, as it gives us enough numbers to be able to disaggregate SSA into country of origin. The use of administrative data minimalizes self-reported bias. In addition, several socioeconomic and migration-related characteristics were available giving us the possibility of adjusting for the variables that better explained the use of PHC, although many factors related to health and health care use remains unmeasured and some variables, like reason for migration, were not specified in a sufficient number of participants to be included in the models. Our study had, however, also limitations. Firstly, our data lack information about patients using private clinics in PHC. Although the Norwegian health care system is mostly public and base on a gate-keeper function of the GP, patients already referred to the specialist or attending only private clinics will appear as if they have not been in contact with PHC. Secondly, our HELFO-database does not include patient’s information for elderly residing in the nursing homes, which may explain part of the elderly populations’ low utilization of the PHC services. Last, the diagnoses in our study were based on ICPC-codes registered for administrative claims and not extracted from electronic records. Generally, these claims include only one diagnose disregarding the number of diseases the patient might present and therefore cannot be used for estimating actual prevalences of diseases. However, the ICPC- codes have far been used and recommended to be an adequate and reliable classification system for comparison of groups in primary health care [44].

## Conclusion

Although Somalis, Ethiopia, Eritrea and the Gambians have a similar distribution of diagnosis, differences exist in their use of GP and ER, with immigrants from Somalia using the PHC system more often than the other groups. However, immigrants from Somalia seem to reduce their use of PHC with a longer duration of stay in Norway. Differences among immigrants from the four sub-Saharan countries should be further explored in order to inform policy makers to attain equity in the provision of PHC.

## Additional file

Additional file 1: Logistic regression of migrants’ use of primary health care services by sex, age groups, employment and, length of stay. The supplemental table shows the results of the logistic regressions of immigrant’s from different sub-Saharan African countries (Somalia, Ethiopia, Eritrea and Gambia) and the use of the general practitioner and the emergency room by sex, different age groups, those employed and the immigrants’ length of stay in Norway. (DOCX 15 kb)

## Abbreviations

ACG: Adjusted clinical groups
ENT: Ear nose throat
ER: Emergency room
GP: General practitioner
HELFO: Norwegian health economics administrative database
ICPC: International classification of primary care
MEDC: Major expanded diagnostic clusters
NPR: National population registry
OR: Odds ratio
PHC: Primary health care
SD: Standard deviation
SSA: Sub-Saharan Africa
WHO: World Health Organization
