# Supplementary material for: Differences in primary health care use among sub-Saharan African immigrants in Norway: a register-based study
Source: BMC Health Serv Res. 2017 Jul 28;17:509. doi: 10.1186/s12913-017-2404-z (PMC5534034; doi:10.1186/s12913-017-2404-z)
Supplement: Additional file 1: — Logistic regression of migrants’ use of primary health care services by sex, age groups, employment and, length of stay. The supplemental table shows the results of the logistic regressions of immigrant’s from different sub-Saharan African countries (Somalia, Ethiopia, Eritrea and Gambia) and the use of the general practitioner and the emergency room by sex, different age groups, those employed and the immigrants’ length of stay in Norway. (DOCX 15 kb) [file 12913_2017_2404_MOESM1_ESM.docx]

Supplemental Table

*Logistic regression of migrants’ use of primary health care services by sex, age groups, employment and, length of stay*

|  | Use of General Practitioner | | | | Use of Emergency room | | | |
| --- | --- | --- | --- | --- | --- | --- | --- | --- |
|  | Somalia  OR (95% CI) | Ethiopia  OR (95% CI) | Eritrea  OR (95% CI) | Gambia  OR (95%CI) | Somalia  OR (95% CI) | Ethiopia  OR (95% CI) | Eritrea  OR (95% CI) | Gambia  OR (95% CI) |
| **Sex**  Men  Women | 1.00  1.56 (1.47- 1.64) | 1.00  1.52 (1.36- 1.70) | 1.00  1.71 (1.51- 1.93) | 1.00  1.49 (1.23- 1.81) | 1.00  1.30 (1.21- 1.39) | 1.00  1.23 (1.06- 1.42) | 1.00  1.27 (1.05- 1.54) | 1.00  1.27 (0.98- 1.64) |
| **Age groups**  **(years)**  0-14  15-44  45-64  >65 | 1.00  1.37 (1.29- 1.46) 2.06 (1.83- 2.32)  2.00 (1.49- 2.70) | 1.00  1.00 (0.87- 1.15)  1.27 (1.02- 1.59)  3.66 (1.06-12.60) | 1.00  0.92 (0.79- 1.07)  1.51 (1.21- 1.89)  1.31 (0.68-2.52) | 1.00  1.15 (0.91- 1.46)  2.15 (1.54- 3.01)  - | 1.00  0.94 (0.87- 1.02)  0.90 (0.77- 1.04)  1.09 (0.77- 1.54) | 1.00  0.71 (0.59- 0.86)  0.58 (0.42- 0.79)  0.49 (0.11- 2.15) | 1.00  0.63 (0.49- 0.80)  0.70 (0.50- 0.79)  1.23 (0.50- 2.98) | 1.00  1.10 (0.79- 1.53)  1.10 (0.70- 1.74)  - |
| **Length of stay (years)**  0-6  >6 | 1.00  0.60 (0.57- 0.64) | 1.00  1.00 (0.89- 1.25) | 1.00  1.19 (1.04- 1.35) | 1.00  0.77 (0.61- 0.97) | 1.00  0.85 (0.79- 0.91) | 1.00  0.87 (0.75- 1.02) | 1.00  1.02 (0.83- 1.24) | 1.00  0.55 (0.41-0.74) |
| **Employed immigrants** | 1.25 (1.21- 1.30) | 1.43 (1.29- 1.59) | 1.44 (1.29- 1.61) | 1.36 (1.17- 1.58) | 1.09 (1.05- 1.14) | 1.12 (0.99- 1.26) | 1.29 (1.12- 1.49) | 1.09 (0.91- 1.30) |
